# Weak Hypotensive Effect of Chronic Administration of the Dual FAAH/MAGL Inhibitor JZL195 in Spontaneously Hypertensive Rats as Revealed by Area under the Curve Analysis

**DOI:** 10.3390/ijms241310942

**Published:** 2023-06-30

**Authors:** Marek Toczek, Piotr Ryszkiewicz, Patryk Remiszewski, Eberhard Schlicker, Anna Krzyżewska, Hanna Kozłowska, Barbara Malinowska

**Affiliations:** 1 Department of Experimental Physiology and Pathophysiology, Medical University of Białystok, Mickiewicza Str. 2A, 15-222 Białystok, Poland; piotr.ryszkiewicz@umb.edu.pl (P.R.); patryk.remiszewski@umb.edu.pl (P.R.); anna.krzyzewska@umb.edu.pl (A.K.); hanna.kozlowska@umb.edu.pl (H.K.); barbara.malinowska@umb.edu.pl (B.M.); 2 Department of Pharmacology and Toxicology, University of Bonn, Venusberg-Campus 1, 53127 Bonn, Germany; e.schlicker@uni-bonn.de

**Keywords:** JZL195, endocannabinoid, FAAH, MAGL, hypertension, spontaneously hypertensive rats, area under the curve

## Abstract

The enhancement of the endocannabinoid tone might have a beneficial influence on hypertension. Polypharmacology proposes multi-target-directed ligands (MTDLs) as potential therapeutic agents for the treatment of complex diseases. In the present paper, we studied JZL195, a dual inhibitor of the two major endocannabinoid-degrading enzymes, fatty acid amide hydrolase (FAAH) and monoacylglycerol lipase (MAGL), in spontaneously hypertensive rats (SHR) and normotensive Wistar Kyoto rats (WKY). Hemodynamic parameters were assessed in conscious animals via radiotelemetry and tail-cuff methods and then evaluated by the area under the curve (AUC). Single administration of JZL195 induced dose-dependent weak hypotensive and bradycardic responses in SHR but not in WKY. Similarly, its chronic application revealed only a slight hypotensive potential which, however, effectively prevented the progression of hypertension and did not undergo tolerance. In addition, multiple JZL195 administrations slightly decreased heart rate only in WKY and prevented the gradual weight gain in both groups. JZL195 did not affect organ weights, blood glucose level, rectal temperature and plasma oxidative stress markers. In conclusion, chronic dual FAAH/MAGL inhibition prevents the progression of hypertension in SHR without affecting some basal functions of the body. In addition, our study clearly proves the suitability of AUC for the evaluation of weak blood pressure changes.

## 1. Introduction

Arterial hypertension is one of the strongest risk factors for cardiovascular diseases, which still remain the leading cause of death worldwide (e.g., [1,2,3]). Approximately 90% of hypertensive patients suffer from primary hypertension, i.e., not related to renal or endocrine disorders. Unfortunately, despite the plenitude of marketed antihypertensive drugs, which act at different molecular targets, resistance to antihypertensive pharmacotherapy is considered a significant problem. Depending on the local circumstances, about 10 to 20% of hypertensive patients are found resistant which increases cardiovascular risk [1]. Therefore, searching for new compounds, with the ability to effectively lower blood pressure (BP), seems justified.

Cannabinoids (phytocannabinoids, synthetic cannabinoids and endocannabinoids) are being extensively investigated as possible antihypertensive compounds, but their impact on cardiovascular parameters under study is often model-dependent. In our study, we used the well-established rodent model of primary hypertension, i.e., spontaneously hypertensive rats (SHR), with Wistar Kyoto rats (WKY) as their normotensive controls. We focused on the two best-studied endocannabinoids exhibiting strong vasodilatory effects in isolated human and animal arteries, i.e., anandamide (AEA) and 2-arachidonoylglycerol (2-AG), as well as the enzymes involved in their degradation, i.e., fatty acid amide hydrolase (FAAH) and monoacylglycerol lipase (MAGL), respectively (for review, see [4,5]).

Antihypertensive properties of AEA in primary hypertension have been explored in several studies to date. The AEA-induced vasorelaxation in SHR was enhanced in the thoracic aorta [6], but diminished in the mesenteric arterial bed [6] and in small resistance mesenteric arteries [7]. Acute intravenous (*i.v.*) injection of AEA or its stable analog methanandamide (MethAEA) into anesthetized and conscious SHR decreased BP and heart rate (HR) with no or moderate effects in the respective normotensive control animals [8,9,10,11]. Similarly, chronic administration of nanoformulated AEA for 4 weeks in SHR revealed a hypotensive effect [11].

FAAH inhibitors indirectly increase the level of AEA by blocking its enzymatic degradation. The FAAH inhibitor URB597 enhanced the vasodilatory effect of AEA [12] more markedly in SHR than in WKY. Moreover, the *i.v.* infusion of URB597 and/or another FAAH inhibitor, AM3506, or of the endocannabinoid reuptake inhibitors, AM404 and OMDM-2, into anesthetized or conscious SHR reduced BP and HR (as opposed to WKY) [8,9,10] but failed to modify these parameters in other studies with conscious SHR [13,14,15]. URB597 given intraperitoneally (*i.p.*; 1 mg/kg; twice a day) for 2 weeks failed to modify BP and HR in SHR [16]. Despite the lack of an antihypertensive effect of chronic *i.p.* URB597 treatment, it had a mostly positive impact on blood vessels, cardiac performance and metabolism, redox balance or inflammatory processes in different tissues (reviewed in detail in [5]).

In contrast to AEA, the knowledge about the impact of 2-AG on cardiovascular parameters is quite limited and this has much to do with its short half-life [17]. Given *i.v.*, 2-AG decreased BP and increased HR in anesthetized mice probably via its arachidonate metabolite [18]. Importantly, 2-AG released in response to vasoconstrictors (e.g., phenylephrine) acts protectively against enhanced vascular tone via CB_1_ receptor-mediated vasorelaxation; this mechanism has also been determined in mesenteric arteries of SHR [19]. The MAGL inhibitor JZL184 decreased HR, but not BP, in pithed rats [20]; its chronic administration diminished the enhanced BP in a mouse model of the Williams–Beuren syndrome (i.e., a genetic disorder affecting the heart, the face and many other parts of the body; [21]). It is also worth noting that in normotensive rats the 2-AG levels are higher than those of AEA not only in the brain [22], but also in the heart [23,24] and in the blood vessels [19].

Hypertension by itself also affects the components of the endocannabinoid system. The AEA levels in SHR were increased in blood plasma [25] and kidneys [26], compared to the respective normotensive controls. Moreover, in patients with diagnosed hypertension, the AEA plasma level was higher and the AEA transporter activity was lower [25,27]. By contrast, other investigators found lower plasma and cardiac levels of AEA and 2-AG in SHR than in WKY, but at the same time, cardiac FAAH and MAGL levels were found to be higher [8,23]. The AEA and 2-AG levels in the aorta and mesenteric (G3) arteries isolated from SHR were higher than those measured in normotensive WKY control [19].

JZL195 is a highly potent and selective dual FAAH/MAGL inhibitor (IC_50_ values of 2 and 4 nM, respectively; [28]); it may have therapeutic potential as suggested by studies in animal models of pain (e.g., [29,30,31], nausea [32,33], pruritus [34], traumatic brain injury [35], Alzheimer’s disease-like sporadic dementia [36] and allergic airway inflammation [37]. One might assume that the enhancement of the endocannabinoid tone by simultaneous inhibition of FAAH and MAGL might produce more pronounced positive effects on hypertension in comparison to single FAAH inhibition, namely as a result of synergistic actions of AEA and 2-AG. Moreover, dual FAAH and MAGL inhibitors are in line with the novel approach of polypharmacology, which proposes multi-target-directed ligands (MTDLs) as potential therapeutic agents, suitable for the treatment of complex diseases, including hypertension [38,39]. Therefore, the aim of our study is to examine the influence of single and multiple administrations of JZL195 on hemodynamic and other physiological parameters in SHR and their normotensive controls (WKY) to establish the antihypertensive potential of dual FAAH/MAGL inhibition.

## 2. Results

### 2.1. General

**Figure 1:**
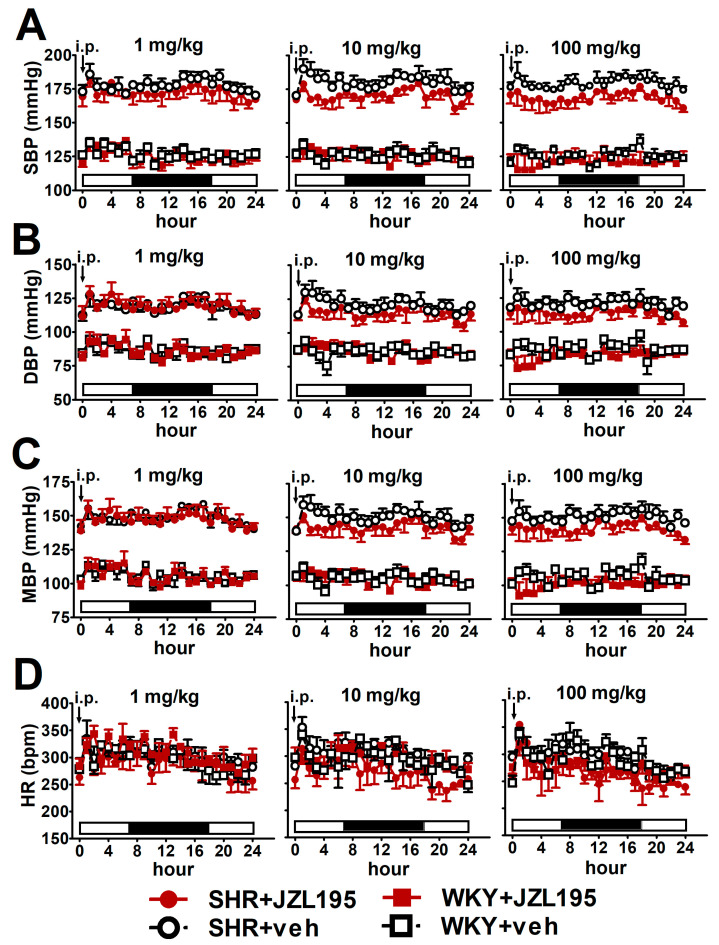
Influence of single administration of JZL195 in increasing doses (1, 10 or 100 mg/kg) or its vehicle on hourly systolic blood pressure (SBP; (**A**)), diastolic blood pressure (DBP; (**B**)), mean blood pressure (MBP; (**C**)) and heart rate (HR; (**D**)) in spontaneously hypertensive rats (SHR) and normotensive Wistar Kyoto rats (WKY) during 24 h after *i.p.* injection. Parameters were measured continuously, radiotelemetrically in unrestrained rats and averaged to give hourly values. The white and black bars at the bottom of the graphs represent the light and dark periods of the day, respectively. Data are expressed as the means ± SEM of 3–4 rats per group. In (**A**–**C**) all values of BP in SHR were higher than their respective values in WKY with *p* < 0.001 (for the sake of clarity, we have not marked the respective significance).

**Figure 2:**
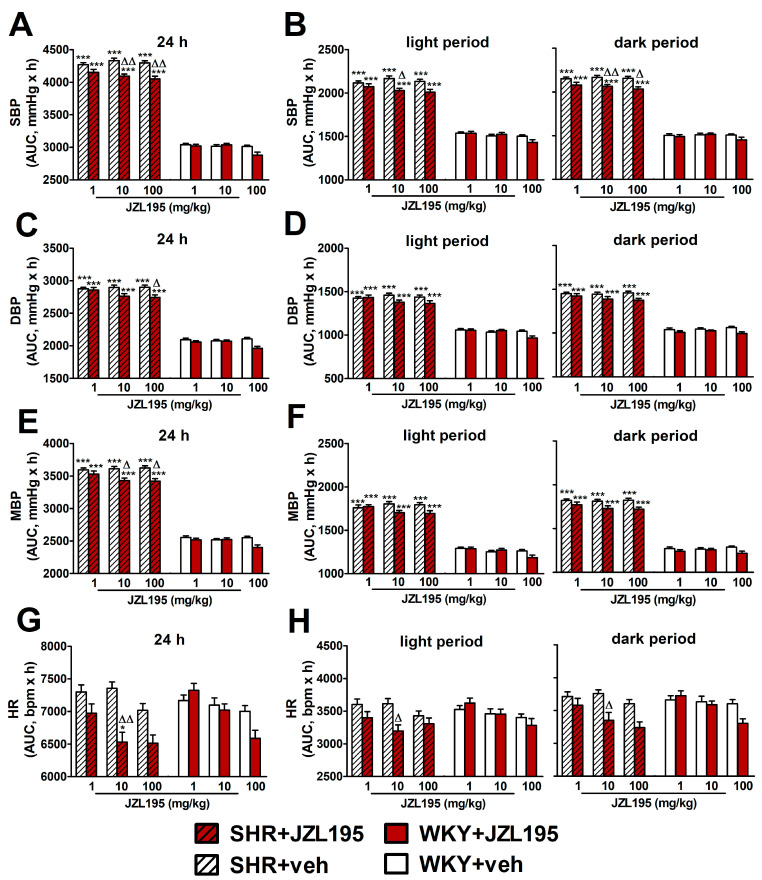
Area under the curve (AUC) for systolic blood pressure (SBP; (**A**,**B**)), diastolic blood pressure (DBP; (**C**,**D**)), mean blood pressure (MBP; (**E**,**F**)) and heart rate (HR; (**G**,**H**)) in spontaneously hypertensive rats (SHR) and normotensive Wistar Kyoto rats (WKY) measured continuously, radiotelemetrically in unrestrained rats during 24 h after *i.p.* injection of JZL195 in increasing doses (1, 10 or 100 mg/kg) or its vehicle. Results are presented for the entire 24 h period (**A**,**C**,**E**,**G**) and for the 12 h light and 12 h dark periods (**B**,**D**,**F**,**H**) of the day. Data are expressed as the means ± SEM of the AUC of 3–4 rats per group; * *p* < 0.05; *** *p* < 0.001 vs. normotensive control (WKY + veh); ^Δ^ *p* < 0.05; ^ΔΔ^ *p* < 0.01 vs. the respective vehicle group.

**Figure 3:**
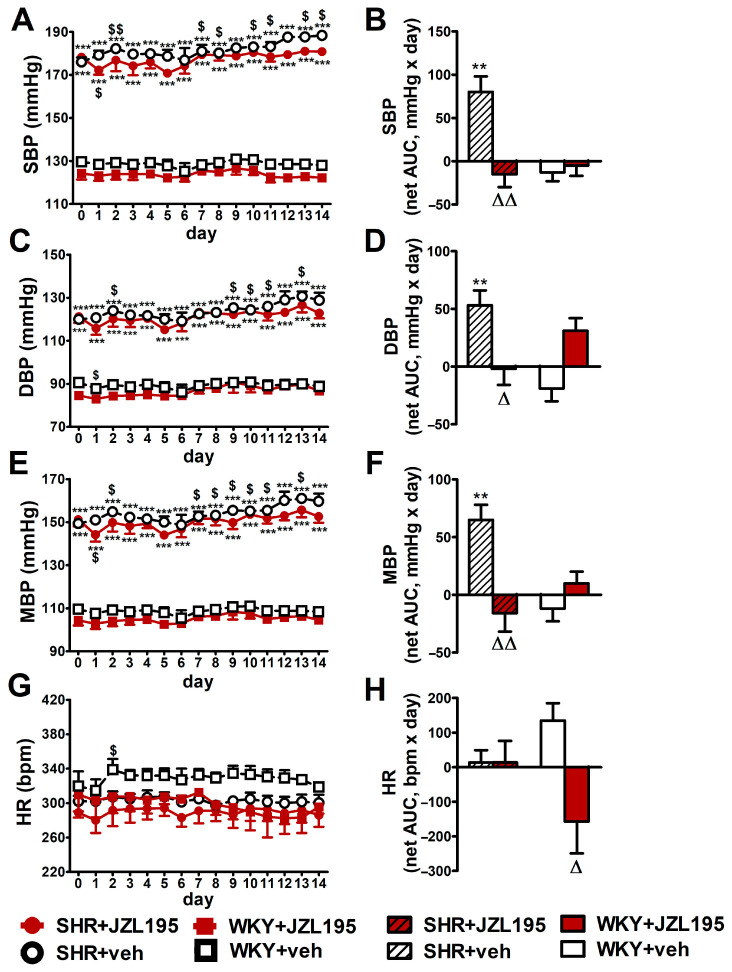
Influence of chronic administration of JZL195 (10 mg/kg, once daily, for 14 days) or its vehicle on systolic blood pressure (SBP; (**A**,**B**)), diastolic blood pressure (DBP; (**C**,**D**)), mean blood pressure (MBP; (**E**,**F**)) and heart rate (HR; (**G**,**H**)) in spontaneously hypertensive rats (SHR) and normotensive Wistar Kyoto rats (WKY). Parameters were measured continuously, radiotelemetrically in unrestrained rats and averaged to give daily values. Data are expressed as the means ± SEM of the daily values (**A**,**C**,**E**,**G**) or net area under the curve (net AUC; (**B**,**D**,**F**,**H**)) of 5–6 rats per group; ** *p* < 0.01; *** *p* < 0.001 vs. normotensive control (WKY + veh); ^Δ^ *p* < 0.05; ^ΔΔ^ *p* < 0.01 vs. respective vehicle group; ^$^ *p* < 0.05; ^$$^ *p* < 0.01 vs. day 0.

**Figure 4:**
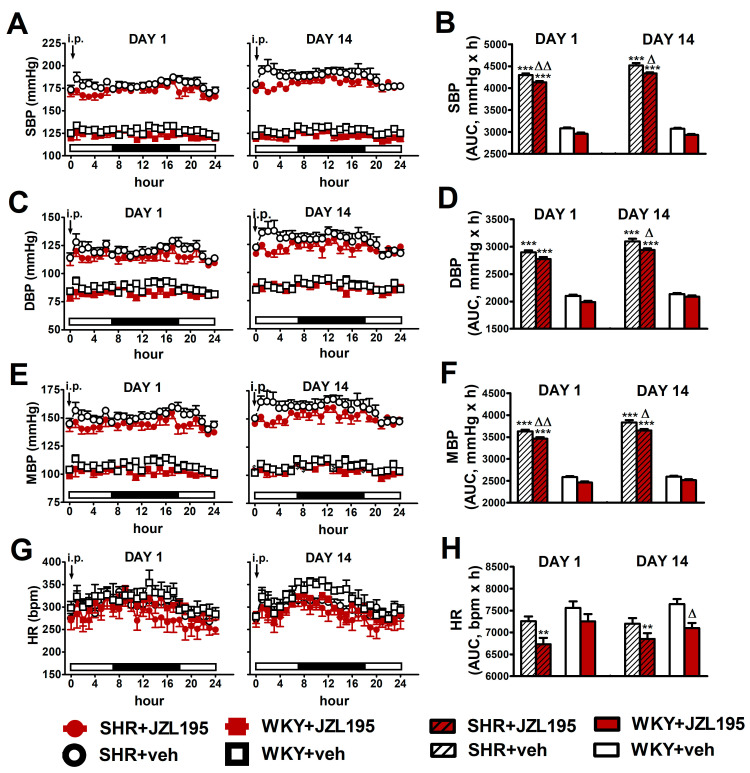
Influence of the 1st (DAY 1) and 14th (DAY 14) administration of JZL195 (10 mg/kg) or its vehicle on systolic blood pressure (SBP; (**A**,**B**)), diastolic blood pressure (DBP; (**C**,**D**)), mean blood pressure (MBP; (**E**,**F**)) and heart rate (HR; (**G**,**H**)) in spontaneously hypertensive rats (SHR) and normotensive Wistar Kyoto rats (WKY) during 24 h after *i.p.* injection. Parameters were measured continuously by radiotelemetry in unrestrained rats and averaged to give hourly values. The white and black bars at the bottom of the graphs represent light and dark periods of the day, respectively. Data are expressed as the means ± SEM of the hourly values (**A**,**C**,**E**,**G**) or area under the curve (AUC; **B**,**D**,**F**,**H**) of 5–6 rats per group; ** *p* < 0.01; *** *p* < 0.001 vs. normotensive control (WKY + veh); in (**A**,**C**,**E**) all values of BP in SHR were higher than their respective values in WKY with *p* < 0.001 (for the sake of clarity, we have not marked the respective significance); ^Δ^ *p* < 0.05; ^ΔΔ^ *p* < 0.01 vs. respective vehicle group.

Average daily values of systolic blood pressure (SBP), diastolic blood pressure (DBP) and mean blood pressure (MBP) measured via the radiotelemetric method before treatment with JZL195 or its vehicle (expressed as absolute values and area under the curve [AUC]) in conscious unrestrained rats were higher (*p* < 0.001) in SHR than their respective values in WKY (Figure 1, Figure 2, Figure 3 and Figure 4). In animals in which dose-dependent effects of JZL195 were determined, the mean daily SBP, DBP and MBP before the experiments were 176 ± 5, 121 ± 4 and 150 ± 4 mmHg in SHR + JZL195 (n = 4); 178 ± 2, 120 ± 0 and 150 ± 1 mmHg in SHR + veh (n = 4); 125 ± 1, 85 ± 1 and 105 ± 1 mmHg in WKY + JZL195 (n = 3) and 128 ± 5, 88 ± 1 and 107 ± 3 mmHg in WKY + veh (n = 3), respectively. By contrast, we did not find any differences in the daily basal HR between these particular groups. Values amounted to 281 ± 10, 290 ± 9, 298 ± 9 and 289 ± 7 beats/min in SHR + JZL195 (n = 4), SHR + veh (n = 4), WKY + JZL195 (n = 3), and WKY + veh (n = 3), respectively.

**Figure 5:**
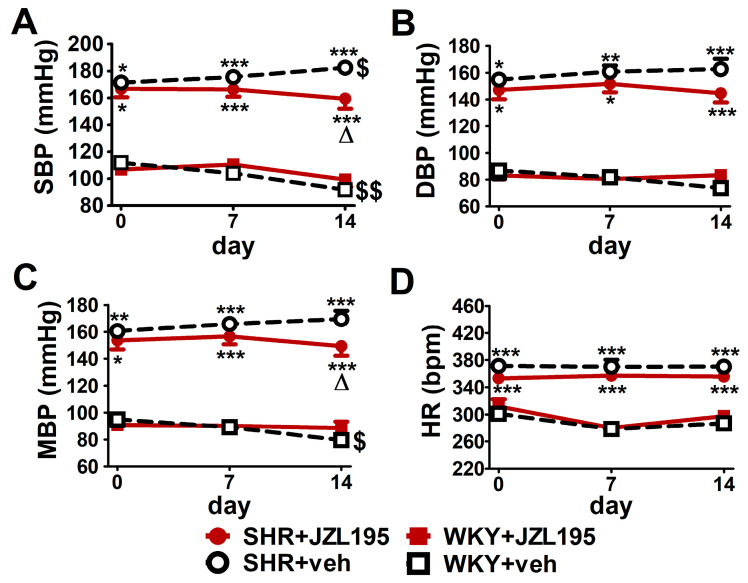
Influence of chronic administration of JZL195 (10 mg/kg, once daily, for 14 days) or its vehicle on systolic blood pressure (SBP; (**A**)), diastolic blood pressure (DBP; (**B**)), mean blood pressure (MBP; (**C**)) and heart rate (HR; (**D**)) in spontaneously hypertensive rats (SHR) and normotensive Wistar Kyoto rats (WKY). Parameters were measured via the tail-cuff method in restrained rats before the first injection and 7 and 14 days after administration of JZL195 or its vehicle (24 h after *i.p.* injection). Data are expressed as the means ± SEM of six rats per group; * *p* < 0.05; ** *p* < 0.01; *** *p* < 0.001 vs. normotensive control group (WKY + veh); ^Δ^ *p* < 0.05 vs. respective vehicle group; ^$^ *p* < 0.05; ^$$^ *p* < 0.01 vs. day 0.

As shown in Figure 5, SBP, DBP and MBP before the first dose of JZL195 or its vehicle measured in conscious restrained rats via the tail-cuff method were higher in SHR than in WKY (by about 55%, 70% and 75%, respectively). In contrast to the results obtained with the radiotelemetric method, HR determined by the tail-cuff method was higher by about 20% in SHR than in WKY before JZL195 treatment. We did not detect any differences in BP and HR between the two SHR and between the two WKY groups before the administration of JZL195 or its vehicle.

### 2.2. Influence of Single Administration of JZL195 (1, 10 and 100 mg/kg) on BP and HR in Conscious Unrestrained SHR and WKY Measured via the Radiotelemetry Method

To determine the cardiovascular effects of increasing doses of JZL195 (1, 10 and 100 mg/kg) or its vehicle on the hemodynamic parameters in SHR and WKY measured via the radiotelemetric method in conscious unrestrained animals for 24 h after injection, we expressed BP and HR as their absolute values (Figure 1), as well as AUC (Figure 2). In SHR, JZL195 decreased all cardiovascular parameters in a dose-dependent manner, which was clearly visible when AUCs for these parameters were compared. JZL195 at doses of 10 and 100 mg/kg reduced SBP and MBP measured over a time period of 24 h maximally by about 5–6% of the AUC compared with the respective vehicle groups. DBP was diminished and tended to be reduced by doses of 100 and 10 mg/kg, respectively, in both cases by about 5%. BP in WKY was not affected markedly by JZL195; however, its highest dose tended to decrease this parameter (Figure 1A–C and Figure 2A,C,E). HR was reduced or tended to be lower in SHR injected with JZL195 (1, 10 and 100 mg/kg) maximally by about 10% of the AUC. A similar tendency was observed in WKY treated with JZL195 at the highest dose (Figure 1D and Figure 2G). The period of the day did not significantly influence the changes in hemodynamic parameters induced by JZL195 (Figure 2B,D,F,H).

### 2.3. Influence of Chronic Administration of JZL195 (10 mg/kg) on BP and HR in Conscious Unrestrained SHR and WKY Measured via the Radiotelemetry Method

Figure 3 presents changes in cardiovascular parameters (expressed as absolute values and as net AUC) measured in conscious unrestrained animals by the radiotelemetric method registered during 14 days of chronic administration of JZL195 (10 mg/kg once daily) or its vehicle. BP was higher in SHR than in WKY throughout the whole experiment. SBP, DBP and MBP (expressed as absolute values in mmHg [Figure 3A,C,E] and as net AUC [Figure 3B,D,F]) increased progressively in SHR during two weeks of vehicle administration, in each case by about 7%. Chronic treatment with JZL195 prevented the above BP increases; this is clearly visible when a net AUC assessment was applied (Figure 3B,D,F). On the other hand, BP was stable in WKY during the two weeks of study and JZL195 did not influence this parameter.

In contrast to BP, HR in SHR was not changed significantly in comparison to baseline (day 0) over the course of the experiment in the vehicle and JZL195 groups (Figure 3G,H). In WKY, vehicle tended to raise and JZL195 tended to decrease HR (Figure 3G,H).

As shown in Figure 4, JZL195 affected hemodynamic parameters similarly on day 1 and day 14, both in SHR and WKY. Moreover, results obtained on the first day confirm our previous observations (presented in Figure 2) that a single JZL195 dose of 10 mg/kg reduced AUC for SBP, DBP, MBP and HR (note that experiments were performed on different groups of rats).

### 2.4. Influence of Chronic Administration of JZL195 (10 mg/kg) on BP and HR in Conscious Restrained SHR and WKY Measured via the Tail-Cuff Method

As shown in Figure 5, like in the conscious unrestrained SHR treated with the vehicle for JZL195, BP tended to rise (by about 5–10%) throughout the 14 days of the experiment. Chronic JZL195 administration did not only stop the progression of hypertension but also tended to gradually reduce BP. After 2 weeks of its application SBP, DBP and MBP were lower by about 12% compared to vehicle-treated SHR. In WKY, BP decreased (SBP) or tended to decrease (DBP and MBP) throughout the two weeks of the experiment by about 15–20%. JZL195 did not affect BP in WKY and HR in SHR and WKY.

### 2.5. Influence of Chronic Administration of JZL195 (10 mg/kg) on Body and Organ Weight, Blood Glucose Level, Rectal Temperature and Oxidative Stress Markers in SHR and WKY

**Figure 6:**
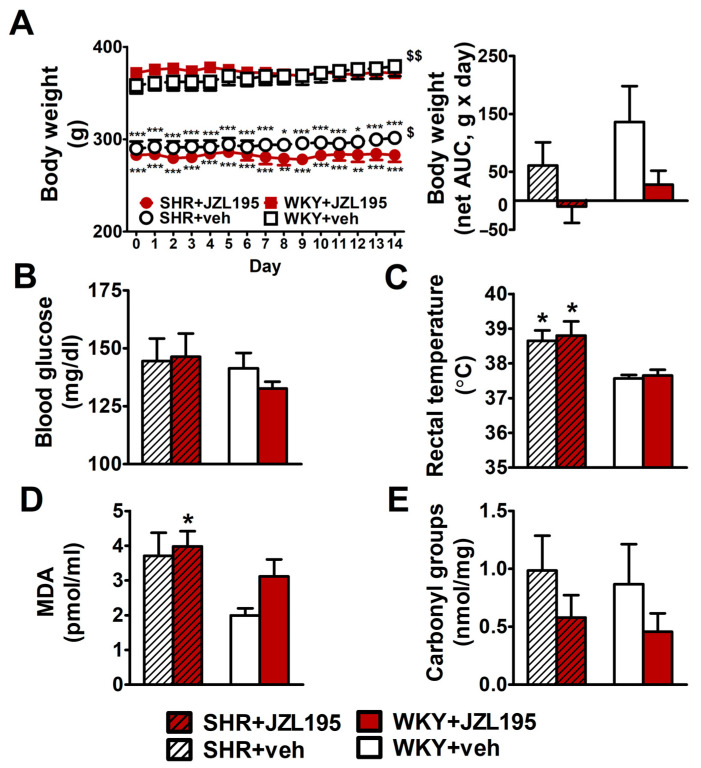
Influence of chronic administration of JZL195 (10 mg/kg, once daily, for 14 days) or its vehicle on body weight (**A**), blood glucose level (**B**), rectal temperature (**C**), plasma malondialdehyde (MDA)-protein adducts (**D**) and carbonyl groups that are created by the oxidation of proteins (**E**) in spontaneously hypertensive rats (SHR) and normotensive Wistar Kyoto rats (WKY). Body weight was measured once daily during the whole experiment and the other parameters were measured at the end of the study. Data are expressed as the means ± SEM of six rats per group; * *p* < 0.05; ** *p* < 0.01; *** *p* < 0.001 vs. normotensive control group (WKY + veh). ^$^ *p* < 0.05; ^$$^ *p* < 0.01 vs. day 0.

Hypertensive animals had a lower body weight and final tibia length (by about 20% and 10%, respectively) than age-matched WKY (Figure 6A and Table 1). The chronic administration of JZL195 for 2 weeks did not influence these parameters significantly. Yet, the small gradual weight gain in SHR and WKY (by about 10 and 20 g during 14 days, respectively) was prevented by JZL195 (Figure 6A,B). Organ weights, expressed as the ratios to final body weight or tibia length, are shown in Table 1. Hypertensive rats exhibited cardiac hypertrophy and more specifically, left ventricle (with septum) hypertrophy; compared to WKY, left ventricle (plus septum weight) was higher by about 30–35% (when its ratio to final body weight was considered) and tended to be higher by about 10–20% (when its ratio to tibia length was considered). The weights of kidneys, liver and lungs were similar in SHR and WKY. The only exception was a lower kidney mass (by about 12–14%) in SHR when it was expressed as a tibia length ratio. Two-week administration of JZL195 did not change organ weights in any group. Final blood glucose levels (Figure 6B) were similar in SHR and WKY, whereas rectal temperature (Figure 6C) was higher in SHR. Chronic administration of JZL195 did not change these parameters.

As shown in Figure 6D,E, MDA, the marker of lipid peroxidation, tended to be higher by about 90% in SHR than in WKY. Yet, the levels of protein carbonyls (markers of protein oxidation) were comparable in control SHR and WKY. JZL195 administration did not affect the MDA level in SHR but tended to increase this parameter in WKY by about 35%. Moreover, it tended to reduce the level of carbonyl groups by about 50% both in SHR and in WKY.

## 3. Discussion

The aim of the present study was to examine the possible antihypertensive properties of single and multiple administrations of the dual FAAH/MAGL inhibitor JZL195. Experiments were performed on spontaneously hypertensive rats, the most common animal model of human primary hypertension [3], in which most antihypertensive drugs are active [40]. Dual FAAH/MAGL inhibitors follow the new trend in contemporary pharmacology of multi-target-directed ligands as therapeutic compounds suitable for the treatment of complex diseases, including hypertension [38,39].

We used two methods for the determination of cardiovascular parameters in conscious rats. Radiotelemetry allows continuous registration of cardiovascular parameters in freely-moving animals. The tail-cuff method allows discontinuous registration only and, in addition, is associated with stress for the rats due to their immobilization in a special holder. Interestingly, the potential hypotensive effect of the phytocannabinoid cannabidiol (CBD) may increase under stress both in preclinical and clinical studies [41]. The use of the tail-cuff method also allowed us to compare the influence of JZL195 with that of the FAAH inhibitor URB597 (previous studies listed in the Introduction) before and 7 and 14 days after their administration.

Both under radiotelemetry and the tail-cuff method, BP was higher in SHR than in WKY. By contrast, although basal HR values, determined radiotelemetrically, were comparable in SHR and WKY, HR was higher in SHR than in WKY when the tail-cuff method was applied. A similar influence of restraint on basal HR values was described in previous studies [24,42].

### 3.1. Effects of Single Administration of JZL195 on Hemodynamic Parameters

Twenty-four-hour radiotelemetry measurement showed only a slight influence of JZL195 on cardiovascular parameters after its *i.p*. administration at doses of 1, 10 and 100 mg/kg. So, we next applied AUC analysis, which has the potential to disclose differences even if they are hardly detectable with other statistical methods [43]. Moreover, AUC analysis is suggested to be the best alternative for the evaluation of the behavior of the arterial pressure over a 24-h period [44].

Indeed, the AUC method allowed us to show a slight but clear dose-dependent decrease in BP and HR, observed during the 24 h after JZL195 administration in SHR but not in WKY (although the highest dose of JZL195 tended to decrease HR in WKY). In our study, we did not observe any influence of the time of the day (light/dark period) on the hemodynamic profile of JZL195.

It has been shown previously that a single intravenous (*i.v.*) administration of the FAAH inhibitors URB597 [8] and AM3506 [9] to pentobarbital-anesthetized SHR resulted in a marked drop in BP to normotensive values. In addition, the more potent inhibitor AM3506 reduced HR. Hypotensive and bradycardic responses to AM3506 in conscious SHR lasted up to an hour [9]. None of the FAAH inhibitors affected the hemodynamic parameters in normotensive WKY. On the other hand, the MAGL inhibitor JZL184 reduced HR but not BP in normotensive pithed rats, i.e., animals in which the destruction of the central nervous system by a steel rod allowed to study the peripheral effects only [20]. We are not able to directly compare the acute cardiovascular effects of JZL195 with those determined for single FAAH or MAGL inhibitors since JZL195 was given *i.p.* and the previously studied FAAH or MAGL inhibitors *i.v*. To the best of our knowledge, the influence of MAGL or dual FAAH/MAGL inhibition on cardiovascular parameters in SHR has so far not been studied.

### 3.2. Effects of Chronic Administration of JZL 195 on Hemodynamic Parameters

In chronic experiments, we examined only one dose of JZL195, i.e., 10 mg/kg *i.p.* once daily for 14 days, which slightly diminished BP and HR in SHR without affecting both parameters in WKY. Based on a body surface area dose translation [45], the 10 mg/kg dose in rats correlates to 20 mg/kg in mice [28]. In mice, the highest brain levels of AEA and 2-AG were determined about 2–4 h after JZL195 20 mg/kg *i.p.*, but significant inhibition of FAAH and MAGL activities still occurred even 24 h after its injection [28]. In rats, JZL195 10 mg/kg *i.p.* significantly enhanced depolarization-induced increases in AEA and 2-AG levels in the nucleus accumbens [46], increased the brain AEA level and attenuated anticipatory nausea [32] and produced an antinociceptive effect in mechanically evoked visceral pain [30]. In mice, chronic administration of JZL195 (20 mg/kg; *i.p.*) for 15 days reversed the biochemical anomalies of streptozotocin-induced Alzheimer’s disease-like sporadic dementia [36].

Basal BP rose in SHR (but not in WKY) during the two weeks of the experiment, as determined under two different methods of BP measurement. Combined use of radiotelemetry and AUC analysis clearly showed that administration of JZL195 (10 mg/kg; *i.p.*) for two weeks prevented the above progression of hypertension in SHR without affecting the HR. In addition to total AUC, we have applied net AUC (the baseline was day 0) to present the effects of chronic administration of JZL195 on hemodynamic parameters and body weight, i.e., two parameters that increased in vehicle groups over the course of the experiment. The use of net AUC allowed us a better evaluation of the chronic JZL195 effects on the parameters under study. In addition, net AUC more readily shows the opposite influences of the vehicle and JZL195 on the parameters under study.

Importantly, the results obtained via the tail-cuff method also revealed that chronic treatment with JZL195 for 2 weeks not only prevented the progression of hypertension but also reduced BP in SHR by about 10–12%; this may be related to the stress connected with the tail-cuff method. Interestingly, as mentioned above, a hypotensive effect of another drug (the phytocannabinoid cannabidiol) occurred under stress conditions both in humans and in experimental animals [41]. In our recent study [16], in which BP was determined with the tail-cuff method only, a 2-week administration of the FAAH inhibitor URB597 did not affect BP and HR in SHR, but reduced BP in the deoxycorticosterone acetate (DOCA)-salt model of secondary hypertension [16]. Thus, we cannot exclude a stronger antihypertensive effect of a dual FAAH/MAGL inhibitor in other models of hypertension. In the only publication describing the effects of chronic administration of a MAGL inhibitor, JZL184 (8 mg/kg; *i.p.*) given for 10 days normalized the enhanced SBP determined in a mouse model for the Williams-Beuren syndrome [21].

Both AEA and 2-AG are known to produce strong vasodilatory effects (see Introduction). The question arises, why does the dual inhibition of FAAH and MAGL, enzymes responsible for AEA and 2-AG degradation, produce such a weak hypotensive response? In our review, we suggested that multitarget vasodilatory (endo)cannabinoids (including AEA and 2-AG that act not only via CB_1_ and CB_2_ receptors) are not effective as antihypertensive compounds after their chronic administration, because they also induce responses increasing BP (e.g., CB_1_ receptors have a pro-oxidant and pro-inflammatory effect and stimulation of central CB_1_ receptors produces a pressor response; for details, see [5]). Thus, we suppose that JZL195 may lead to both depressor and pressor effects and consequently only to a slight decrease in BP.

Analysis of the 24-h hemodynamic profile during the first and the last day of JZL195 treatment allows us to exclude the development of tolerance, sometimes observed for MAGL inhibition [22]. Tolerance did also not develop with respect to the antiallodynic effects of JZL195 given subcutaneously to mice (3 and 18 mg/kg for 5 days) [29]. However, the authors of the latter study did not exclude that tolerance might occur at higher doses of JZL195, since they used JZL195 at doses that were likely to produce submaximal increases in the levels of AEA and 2-AG only. The same might also be true in the case of our experiments.

JZL195 failed to affect BP in WKY, regardless of whether BP was determined by radiotelemetry or by the tail-cuff method. A lack of effect or a weak fall in BP only is typical for the majority of antihypertensive compounds and is related to the low basal BP level in normotensive animals.

Cannabinoids produce tachycardia in humans, but bradycardia in anesthetized and conscious experimental animals (for review, see [47]). The FAAH inhibitor AM3506 decreased HR in anesthetized and conscious SHR, but not in WKY [9]. In our hands, dose-dependent weak bradycardia (or a tendency to decrease HR) was determined in SHR in the case of AUC for single JZL195 administration and in WKY in the case of AUC for the highest dose of single JZL195 administration and for chronic application of JZL195. However, JZL195 did not change HR in SHR or WKY when the tail-cuff method was used. Moreover, chronic administration of JZL195 failed to improve cardiac hypertrophy associated with hypertension in SHR. Similarly, URB597 given for two weeks did not diminish cardiac hypertrophy in SHR [16], whereas chronic JZL184 (8 mg/kg) administration for 10 days diminished the cardiac hypertrophy in a mouse model for Williams-Beuren syndrome [21].

### 3.3. The Influence of Chronic Administration of JZL195 on Body and Organ Weights, Blood Glucose Level, Rectal Temperature and Plasma Oxidative Stress Markers

As mentioned in the Introduction, the increase in endocannabinoid tone by inhibition of FAAH and MAGL represents a promising therapeutic approach in various diseases. Such a strategy allows minimizing the undesirable side effects, associated mainly with the direct stimulation of cannabinoid CB_1_ receptors [22,48]. Unfortunately, the first clinical trial (January 2016) of the FAAH inhibitor BIA 10-2474 led to the death of one volunteer and to the hospitalization of another four (for review, see [22,48]). Thus, the additional aim of our study was a search for potential side effects of chronic FAAH/MAGL inhibition.

SHR had lower body weight and tibia length, but higher rectal temperature and a tendency to higher levels of MDA in plasma or carbonyl groups in the heart (markers of lipid peroxidation and oxidative damage to proteins, respectively), as shown previously [23,24,49,50]. On the other hand, blood glucose levels, as well as kidney, liver and lung weights were comparable in SHR and WKY.

In our hands, chronic administration of the dual FAAH/MAGL inhibitor for 2 weeks caused only one, but not significant, visible effect apart from the modification of hemodynamic parameters. It prevented the gradual weight gain in SHR and in WKY. In another study [51], repeated daily injections of JZL195 (8 mg/kg) to mice significantly increased 2-h food intake, but did not increase 24-h food intake during a 2-week treatment period. CB_1_ and CB_2_ receptors have opposing effects on body weight. The best-known phytocannabinoid Δ^9^-tetrahydrocannabinol (Δ^9^-THC) increases body weight via central and peripheral CB_1_ receptors; consequently, inverse agonism at the CB_1_ receptor leads to a decrease. The selective CB_1_ receptor antagonist/inverse agonist rimonabant was marketed for obesity treatment in Europe in 2006 and withdrawn in 2008 [52]. CB_2_ receptor stimulation leads to a decrease in body weight [52].

Chronic JZL195 administration, both to SHR and WKY, did not modify kidney, liver and lung weights, rectal temperature, blood glucose levels and plasma levels of two markers of oxidative stress, i.e., MDA (marker of lipid peroxidation) and protein carbonyl groups (marker of protein oxidation). However, it tended to reduce the level of protein carbonyls both in SHR and in WKY. On the other hand, it tended to increase the MDA level in WKY. Similarly, we found previously that chronic URB597 administration increased plasma MDA levels in WKY [23].

Overall, in terms of the studied physiological parameters, chronic administration of the dual FAAH/MAGL inhibitor JZL195 seems to be safe for both hypertensive and normotensive animals. Thus, we confirmed and extended the previous observations, that mice treated chronically with JZL195 (20 mg/kg; *i.p.* for 6 days) did not show any overt signs of toxicity or lethality [28].

### 3.4. Limitations of the Study

In the present study, we have examined the potential hypotensive effects of a single and a two-week administration of the dual FAAH/MAGL inhibitor JZL195 on BP and HR in SHR. Other results may be obtained if JZL195 is administered at a higher dose or for a longer time. Moreover, additional research is needed to investigate the changes in plasma endocannabinoid levels by JZL195; at the moment, we can only assume that its effects are mediated through the increase of the endocannabinoid tone. Furthermore, the use of other models of experimental hypertension may lead to different results, e.g., as mentioned above, chronic administration of the FAAH inhibitor URB597 did not affect BP in SHR, but reduced it in the deoxycorticosterone acetate (DOCA)-salt model of secondary hypertension [16].

## 4. Materials and Methods

### 4.1. Animals

All surgical procedures and experimental protocols were performed in accordance with European and Polish legislation and were approved by the local Animal Ethics Committee in Olsztyn (Poland; decision no. 50/2016 from 21 December 2016). The study was carried out in compliance with the Three Rs principle (reduction, replacement and refinement). Rats were obtained from the Center of Experimental Medicine of the Medical University of Białystok (Poland). Experiments were performed on male SHR and normotensive control rats (WKY). Prior to the experiments, SBP was measured non-invasively in all rats. Only SHR with SBP ≥ 150 mmHg and WKY with SBP < 150 mmHg were included in the study. Animals had free access to tap water and food pellets and were housed in a temperature (22 ± 1 °C) and humidity (55 ± 5%) controlled room under a 12/12 h light/dark cycle.

### 4.2. Experimental Groups and Protocol

Rats were allocated to the following four groups:

1. SHR + JZL195—hypertensive rats treated with JZL195 (1, 10 or 100 mg/kg, *i.p.*, corresponding to 2.3 × 10^−4^, 2.3 × 10^−5^ and 2.3 × 10^−6^ mol/kg, respectively);
2. SHR + vehicle (veh)—hypertensive rats treated *i.p.* with vehicle for JZL195;
3. WKY + JZL195—normotensive rats treated with JZL195 (1, 10 or 100 mg/kg; *i.p.*);
4. WKY + veh—normotensive rats treated *i.p.* with vehicle for JZL195.

The body mass of rats in all experiments was measured before each injection and at the end of the experiments. Animals were 9–10 weeks old at the beginning of JZL195/vehicle treatment.

In the first set of experiments, we investigated the effectiveness of JZL195 in increasing doses (1, 10 or 100 mg/kg) or its vehicle on hemodynamic parameters measured using radiotelemetry transmitters in conscious unrestrained SHR and WKY. Each animal was injected with JZL195 in increasing doses or with vehicle for the following three days.

In the next set of experiments, we analyzed the effects of chronic administration of JZL195 (10 mg/kg) or its vehicle on hemodynamic parameters measured continuously using radiotelemetry transmitters in conscious unrestrained SHR and WKY. Rats were injected with JZL195 or vehicle every 24 h for 14 days. Twenty-four hours after the last injection, rats were sacrificed.

In the last set of experiments, we investigated the influence of chronic administration of JZL195 (10 mg/kg) on hemodynamic parameters, body and organ weights, rectal temperature and biochemical parameters in the blood. Rats were administered with JZL195 or its vehicle every 24 h for 14 days. Hemodynamic parameters were measured using the tail-cuff method in conscious restrained rats before the first dose and after 7 and 14 days (24 h after the last injection). At the end of the experiments, rats were anesthetized with pentobarbital sodium (70 mg/kg; *i.p.*) and then blood glucose, rectal temperature and tibia length were measured, blood samples were collected and internal organs were weighed.

### 4.3. Measurement of BP and HR in Conscious Unrestrained Rats via the Radiotelemetry Method

As described previously [24], implantable radiotelemetric transmitters (HD-S10; Data Sciences International, St. Paul, MN, USA) were used to measure BP (SBP, DBP, MBP) and HR in conscious unrestrained rats. Briefly, rats were anesthetized with pentobarbital sodium *i.p.* (70 mg/kg; i.e., ~300 μmol/kg). Then, catheters were implanted into the femoral artery, and the body of the radiotransmitter was placed into a subcutaneous pocket. Rats were allowed to recover for 1 week before the measurements. The telemetric implants sent signals to receivers located under the cages in which animals were individually housed. Then the receivers sent the data to the matrix connected to a computer. Hemodynamic parameters were measured continuously 24 h a day during the whole experiment.

### 4.4. Measurement of BP and HR in Conscious Restrained Rats via the Tail-Cuff Method

SBP, MBP and HR were measured using the non-invasive tail-cuff method with the Non-Invasive Blood Pressure Controller (ADInstruments, Sydney, Australia) as described previously [24]. DBP was calculated from the equation DBP = (3 × MBP − SBP)/2. During the measurements, animals were kept in holders, which were placed in automatic heaters at an ambient temperature of 30–34 °C for 20 min. From each rat, a minimum of three recordings of hemodynamic parameters were taken and then averaged.

### 4.5. Measurement of Blood Glucose Level, Rectal Temperature, Organ Weights and Oxidative Stress Markers

At the end of the study, blood glucose levels were measured by a glucometer (Accu-Chek Active; Roche Diagnostics GmbH, Mannheim, Germany) in tail-tip blood samples of anesthetized rats. Rectal temperature was determined using a rectal probe transducer (RDT 100; Bio-Sys-Tech, Białystok, Poland). Heart, kidneys, liver and lungs were excised and then their weights were determined and expressed as organ weight/body weight (organ weight/BW, mg/g) ratio and organ weight/tibia length (organ weight/TL, mg/mm) ratio.

Blood samples were obtained from anesthetized animals by left ventricle puncture and collected into ethylenediaminetetraacetic acid (EDTA) tubes. Then plasma separation from whole blood was carried out by centrifugation at 2000 rpm for 10 min at 4 °C. Commercial enzyme-linked immunosorbent assay (ELISA) kits (Abcam [ab], Cambridge, UK) were used for the colorimetric determination of the level of oxidative stress markers in the rat plasma, i.e., MDA-protein adduct levels were quantitated using the MDA Assay Kit (ab238537) and protein carbonyls (a biomarker of protein oxidation) levels were measured using the Protein Carbonyl ELISA Kit (ab238536).

### 4.6. Drugs

Drugs were obtained from the following sources: (4-nitrophenyl) 4-[(3-phenoxyphenyl)methyl]piperazine-1-carboxylate (JZL195) from MedChemExpress (Monmouth Junction, NJ, USA); ethanol from POCH (Gliwice, Poland); Tween-80 from Sigma-Aldrich (Steinheim, Germany); pentobarbital sodium from Biowet (Puławy, Poland) and NaCl from Chempur (Piekary Śląskie, Poland). Pentobarbital sodium was dissolved in saline and injected in a volume of 2 mL/kg. JZL195 was administered as a milky suspension at a volume of 1 mL/kg (at a dose of 1 and 10 mg/kg) and 2 mL/kg (at a dose of 100 mg/kg). The vehicle for JZL195 consisted of ethanol, Tween-80 and 0.9% saline mixed in a ratio of 3:1:16.

### 4.7. Calculations and Statistical Analysis

According to Burgess et al. [43] and Greaney et al. [53] AUC or net AUC analysis was performed to consider both the size and duration of the changes in the parameters under study. Net AUC was applied to present the effects of chronic administration of JZL195 on hemodynamic parameters and body weight i.e., two parameters that increased in vehicle groups over the course of the experiment. Net AUC was calculated by subtracting negative AUC below baseline from that above (baseline recordings were taken before the treatment, on day 0). Results are expressed as means ± SEM (standard error of the mean; n = number of animals). Statistical analysis was performed using Graph Pad Prism 5 and 9 (GraphPad Software, San Diego, CA, USA). The normality of data distribution was evaluated with the Kolmogorov–Smirnov test. If the data were normally distributed, the paired Student’s t-test was employed to compare the baseline and treatment effect. When the normal distribution of data could not be assumed, we used the Wilcoxon test. Inter-group statistical comparisons were made by one-way analysis of variance (ANOVA) followed by Bonferroni’s multiple comparisons tests. Post hoc analysis was conducted only if F was significant. If the data were not normally distributed, we used the Kruskal–Wallis test with Dunn’s post hoc test to compare multiple groups. Dunn’s test was only used when the Kruskal–Wallis test yielded a significant level. A value of *p* < 0.05 was considered statistically significant.

## 5. Conclusions

Our study is the first one in which the hemodynamic profile of a dual FAAH/MAGL inhibitor was examined. A single administration of JZL195, an MTDL which possesses the above properties, produces dose-dependent weak hypotensive and bradycardic responses in SHR but not in their normotensive controls; the hemodynamic alterations in SHR are independent of the period of the day. Chronic application of JZL195 shows only a slight hypotensive potential, which does not undergo tolerance, but effectively prevents the progression of hypertension and does not produce undesirable effects. Dual FAAH/MAGL inhibitors may become future drugs for the treatment of pain, nausea, pruritus, dementia, allergic airway inflammation and traumatic brain injury (for the literature, see Introduction and [22]). Prevention of the progression of hypertension induced by dual FAAH/MAGL inhibitors might be an additional beneficial property of such compounds when used for other indications. Moreover, our study clearly proves the significant role of AUC registration in the evaluation of weak BP changes.

## Figures and Tables

**Table 1:** Influence of chronic administration of JZL195 (10 mg/kg, once daily, for 14 days) or its vehicle on the organ weights of spontaneously hypertensive rats (SHR) and normotensive Wistar Kyoto rats (WKY).

Parameter		SHR + veh	SHR + JZL195	WKY + veh	WKY + JZL195
Final body weight (BW; g)		283 ± 7 ***	302 ± 5 ***	372 ± 3	379 ± 10
Tibia length (TL; mm)		33.6 ± 0.4 ***	33.7 ± 0.3 ***	37.9 ± 0.3	38.2 ± 1.0
Heart	Heart/BW (mg/g)	3.5 ± 0.3 **	3.5 ± 0.1 **	2.7 ± 0.1	2.7 ± 0.04
	Heart/TL (mg/mm)	29.6 ± 1.6	31.5 ± 1.1 *	26.6 ± 0.7	27.1 ± 0.3
Left ventricle (LV)	LV + septum/BW (mg/g)	2.7 ± 0.3 **	2.7 ± 0.1 **	1.9 ± 0.02	2.0 ± 0.04
	LV + septum/TL (mg/mm)	22.7 ± 1.6	23.9 ± 1.1 **	19.0 ± 0.1	20.1 ± 0.4
Right ventricle (RV)	RV/BW (mg/g)	0.7 ± 0.04	0.7 ± 0.1	0.7 ± 0.1	0.7 ± 0.04
	RV/TL (mg/mm)	6.0 ± 0.3	6.4 ± 0.6	6.6 ± 0.8	6.5 ± 0.4
Kidney	Kidney/BW (mg/g)	3.5 ± 0.1	3.4 ± 0.04	3.5 ± 0.1	3.5 ± 0.04
	Kidney/TL (mg/mm)	29.0 ± 0.4 ***	30.7 ± 0.4 ***	33.9 ± 0.7	35.1 ± 0.3
Liver	Liver/BW (mg/g)	38.3 ± 0.8	37.1 ± 1.5	36.4 ± 0.5	36.4 ± 0.5
	Liver/TL (mg/mm)	323.8 ± 13.4	332.6 ± 14.4	357.1 ± 4.6	361.8 ± 8.1
Lungs	Lungs/BW (mg/g)	4.6 ± 0.1	4.5 ± 0.1	4.1 ± 0.1	4.1 ± 0.2
	Lungs/TL (mg/mm)	38.5 ± 0.8	40.6 ± 1.0	40.0 ± 1.3	40.9 ± 1.7

BW, body weight; LV, left ventricle; RV, right ventricle; TL, tibia length; veh, vehicle. Organ weights were measured at the end of the study and are presented as organ weight/BW ratio and organ weight/TL ratio. Data are expressed as the means ± SEM of six rats per group; * *p* < 0.05; ** *p* < 0.01; *** *p* < 0.001 vs. normotensive control group (WKY + veh).

## Data Availability

The data presented in this study are available on request from the corresponding author. The data are not publicly available due to privacy.
